# Global gene expression analysis of the response of physic nut (*Jatropha curcas* L.) to medium- and long-term nitrogen deficiency

**DOI:** 10.1371/journal.pone.0182700

**Published:** 2017-08-17

**Authors:** Qi Kuang, Sheng Zhang, Pingzhi Wu, Yaping Chen, Meiru Li, Huawu Jiang, Guojiang Wu

**Affiliations:** 1 Key Laboratory of Plant Resources Conservation and Sustainable Utilization, South China Botanical Garden, Chinese Academy of Sciences, Guangzhou, China; 2 Guangdong Provincial Key Laboratory of Applied Botany, South China Botanical Garden, Chinese Academy of Sciences, Guangzhou, China; 3 University of Chinese Academy of Sciences, Beijing 100049, PR China; Universidade de Lisboa Instituto Superior de Agronomia, PORTUGAL

## Abstract

*Jatropha curcas* L. is an important biofuel plant with excellent tolerance of barren environments. However, studies on the regulatory mechanisms that operate in this plant in response to nitrogen (N) shortage are scarce. In this study, genome-wide transcriptional profiles of the roots and leaves of 8-week old physic nut seedlings were analyzed after 2 and 16 days of N starvation. Enrichment results showed that genes associated with N metabolism, processing and regulation of RNA, and transport predominated among those showing alterations in expression. Genes encoding transporter families underwent major changes in expression in both roots and leaves; in particular, those with roles in ammonia, amino acid and peptide transport were generally up-regulated after long-term starvation, while *AQUAPORIN* genes, whose products function in osmoregulation, were down-regulated. We also found that *ASPARA−GINASE B1* and *SARCOSINE OXIDASE* genes were up-regulated in roots and leaves after 2 and 16 d N starvation. Genes associated with ubiquitination-mediated protein degradation were significantly up-regulated. In addition, genes in the JA biosynthesis pathway were strongly activated while expression of those in GA signaling was inhibited in leaves. We showed that four major classes of genes, those with roles in N uptake, N reutilization, C/N ratio balance, and cell structure and synthesis, were particularly influenced by long-term N limitation. Our discoveries may offer clues to the molecular mechanisms that regulate N reallocation and reutilization so as to maintain or increase plant performance even under adverse environmental conditions.

## Introduction

Nitrogen (N) is one of the most essential elements for plant growth, but N supply is often limited by environmental conditions. N limitation affects all aspects of plant function, since N is a key component of amino acids, nucleic acids, chlorophyll, ATP, and several plant hormones [1], [2], [3]. In order to cope with a lack of N, plants have evolved regulatory systems enabling them to adapt to adverse environments. High-throughput platforms such as microarray and RNA sequencing (RNA-seq) are effective methods with which to establish massive sets of transcriptomic, which can be used to delineate integrative networks [4], [5], [6]. Previous analysis of transcriptome profiles in Arabidopsis showed that two days of N deprivation repressed the expression of many genes, including those associated with photosynthesis, chlorophyll synthesis, and plastid protein synthesis, at the same time inducing many genes for secondary metabolism and the reprogramming of mitochondrial electron transport [7]. Another study showed that genes involved in nitrogen assimilation, chlorophyll metabolism, photosynthesis, photorespiration, photosystem (PS), the oxidative pentose-phosphate pathway, and ribosomal components were repressed, while genes participating in the accumulation of starch, protein degradation, and anthocyanin synthesis, together with peroxidase and MYB transcription factors *PAP1* and *PAP2* were significantly up-regulated in Arabidopsis shoots by three weeks of low nitrate treatment [8]. In reproductive stage wheat, transcriptome clustering indicated that the expression of genes encoding NiR and ferredoxin-glutamate synthase, which participate in N assimilation, a protease, fructan-related enzyme, ribulose bisphosphate carbox-ylase/oxygenase (Rubisco) small subunit, two subunits of PSII and enzymes of the jasmonic acid (JA) biosynthesis pathway was elevated, whereas transcription of genes for sucrose synthase, and xyloglucan endotransglucosylase and arabinoxylan arabino-furanohydrolase which function in cell wall modification, was reduced under low N conditions [9]. Meta-analysis that integrated the publicly-available transcriptome data for roots treated with different N concentrations demonstrated that the most robustly nitrate-responsive functions were those of transport, signaling, and metabolism, and that G2-like transcription factors (TFs) were hub genes controlling transport and signaling functions [10]. These results indicated that most genes responsive to N limitation are associated with metabolic pathways for N availability, and that the transport of N-containing components is strongly impacted by N conditions.

Recently, studies have been published concerning the behavior of different organs during periods of exposure to N limitation. After 2 d and 10 d of N starvation in a hydroponic growth system, the genes up-regulated in Arabidopsis roots were found to be clustered in the metal handling, amino acid (AA) metabolism, transport and stress categories at day 10, whereas the similar groups were overrepresented among the genes down-regulated in shoots, except that metal handling, TCA cycle, hormone metabolism, and redox system components were specifically down-regulated at day 10 [11]. In Arabidopsis under 4 d, 7 d and 10 d of N-free treatment, genes commonly up-regulated in true leaves mainly encoded members of the NAC and MYB families of TFs, and enzymes of protein degradation and secondary metabolism, while genes down-regulated at all time points included components of photosystem, protein synthesis, AA synthesis, tetrapyrrole synthesis and nucleotide metabolism [12]. In rice, after 1 h, 1 d and 7 d of N starvation, more kinase-related genes were down-regulated than up-regulated in roots, whereas the opposite was true for shoots, and large numbers of TF-related genes were transiently activated after 1 h stress, in contrast to genes encoding transporters, which were activated under long-term N starvation [13]. It is estimated that the percentage of N responsive genes in the maize transcriptome is similar to that observed in Arabidopsis [14]. Moreover, 88% of the Arabidopsis genes showing a response to given treatment did so in an organ-specific manner [15]. However, although there has been much research on model plants, studies on responses to N limitation in non-model species including trees are scarce, and the integrative networks that operate in such species are still unclear.

*J. curcas* (physic nut), a multipurpose shrub or tree with medical uses and considerable potential as biofuel, which belongs to the family *Euphorbiaceae*, possesses valuable characteristics, such as rapid growth, drought tolerance, and adaptation to a wide range of environmental conditions [16], [17]. We have established a high-density genetic map for further dissection of the molecular mechanisms underlying these traits, based on previous studies on the *J. curcas* genome [18]. Research into the response of physic nut to salt and drought stresses has also been conducted recently [19], [20]. Information about the genome and transcriptomic profiles of physic nut tissues can provide valuable resources not only for fundamental studies but also for practical application in improving the resistance of the species to stresses. However, studies on the responses of physic nut roots and leaves to N depletion have not previously been reported. The work presented here focuses on obtaining detailed insights into the impacts of N starvation on *J. curcas* in different organs and over medium and longer time-frames. We obtained the transcriptomic profiles of roots and leaves after 2 d and 16 d of nitrate deficiency in order to explore the effects of these treatments at a molecular level. Genes showing consistent changes in expression in response to N deficiency were identified, and the adaptive responses potentially associated with these changes are discussed. We aim to elucidate the organ-specific responses of *J. curcas* to medium- and long-term N starvation, and hence provide molecular tools with which to improve the efficiency of N utilization.

## Materials and methods

### Plant material and growth

Seedlings of physic nut cultivar GZQX0401 were grown to the six-leaf stage in pots containing sand irrigated daily with Hoagland nutrient solution [20]. Before stress treatments were applied, the growth medium was washed with deionized water to removing soluble ions. The control seedlings then continued to irrigate by standard nutrient solution whereas the treatments were imposed by watering with N-deficient Hoagland nutrient solution (5 mmol/L CaCl_2_ substituted for 5 mmol/L Ca(NO_3_)_2_ and 5 mmol/L KCl instead of 5 mmol/L KNO3) every day.

On the basis of our previous observation of changes in net photosynthetic rate (Pn) in physic nut leaves under N limitation, seedlings of the control group and the treatment group were sampled at two time points. These were the point at which the Pn ratio began a rapid process of attenuation to 80% after two days of treatment (mid-term N deficiency response), and the time at which ratio of Pn was maintained at 64% after a 16-day treatment (long-term N deficiency response). Root samples comprised all root tips 5-10 mm long, and leaf blades sampled were from the fourth fully expanded leaf from the apex. Samples were harvested from three seedlings for each time point, washed thoroughly with distilled water, and immediately frozen in liquid nitrogen until required for RNA extraction. Independent biological replicates were taken in two consecutive years and analyzed separately.

### RNA preparation and the construction of transcriptome library

RNA was extracted from samples of roots and leaves using the CTAB method [21] with RNase-free DNase I (Roche) remove DNA. The RNA quality was determined using Agilent Bioanalyzer Model 2100. The cDNA libraries were constructed with an Illumina preparation kit following the manufacturer’s protocol and sequenced on an Illumina GAII platform in BGI, Shenzhen (http://www.genomics.cn/index). A preprocessed database of all possible CATG+17 nucleotide tags was created using our genomic reference database (GenBank: AFEW00000000.1). All tags were mapped to the reference databases for annotation; only where there was no more than one nucleotide mismatch per tag were the annotations accepted as valid. TPM, the number of tags aligned to a given gene per million total tags, as calculated according to the formula *TPM* = *C* × 10^6^/*N* (where C means = count of tags that were uniquely aligned to a certain gene, and N = total count of clean tags).

### Criteria for identifying differentially expressed genes (DEGs)

The criteria used to identify DEGs were that they should gave a P<0.01 cut-off using IDEG6 (http://telethon.bio.unipd.it/bioinfo/IDEG6_form/), and ratios of gene expression levels calculated as |log2(treatment TPM/control TPM)| ≥ 1 (represented at least a 2-fold changed in expression level). To improve accuracy, the TPM-value threshold was set at equal to or greater than 5 in order to exclude transcripts expressed very low levels. There were two biological replicates and only changes consistent in both replicates were considered to indicate true DEGs. To determine protein sequence similarities with Arabidopsis TAIR10 accessions, pair-wise BLASTP sequence comparisons were performed. Only alignments with an E-value cut-off of ≤ 1e-5 were analyzed further. Enrichment patterns were clustered by local MapMan 3.6.ORC1 software according to TAIR10 accession numbers. Metabolic pathway analysis was carried out with reference to the Kyoto Encyclopedia of Genes and Genomes (KEGG) (http://www.genome.jp/kegg/pathway.html) and BioCyc Database Collection (http://biocyc.org/ARA/class-tree?object=Pathways) websites. Heatmaps were constructed by MultiExperiment Viewer software package. Sequence data have been deposited in the series accession number SUB2567840 (SRX2721077- SRX2721092).

### Quantitative RT-PCR (RT-qPCR)

To confirm the results of the transcriptomic analysis, RNA was extracted from a separate experiment that had responded to N limitation for RT-qPCT analysis. A total of 1 μg RNA of each sample was used as a template in a 20 μl reverse transcription reaction mix. The transcript level for each gene was measured using a Roche PCR system and a SYBR PrimeScript RT-PCR Kit II as described in a previous report to confirm the results of the transcriptomic analysis [20]. Calculations were carried out using the *δδ* CT method. Transcript levels were quantified relative to the level of the *JcACTIN* transcript as an internal control. The primer sequences used are listed in (S1 Table).

## Results

### Overview of transcriptomic responses in roots and leaves to medium- and long-term N starvation

**Fig 1:**
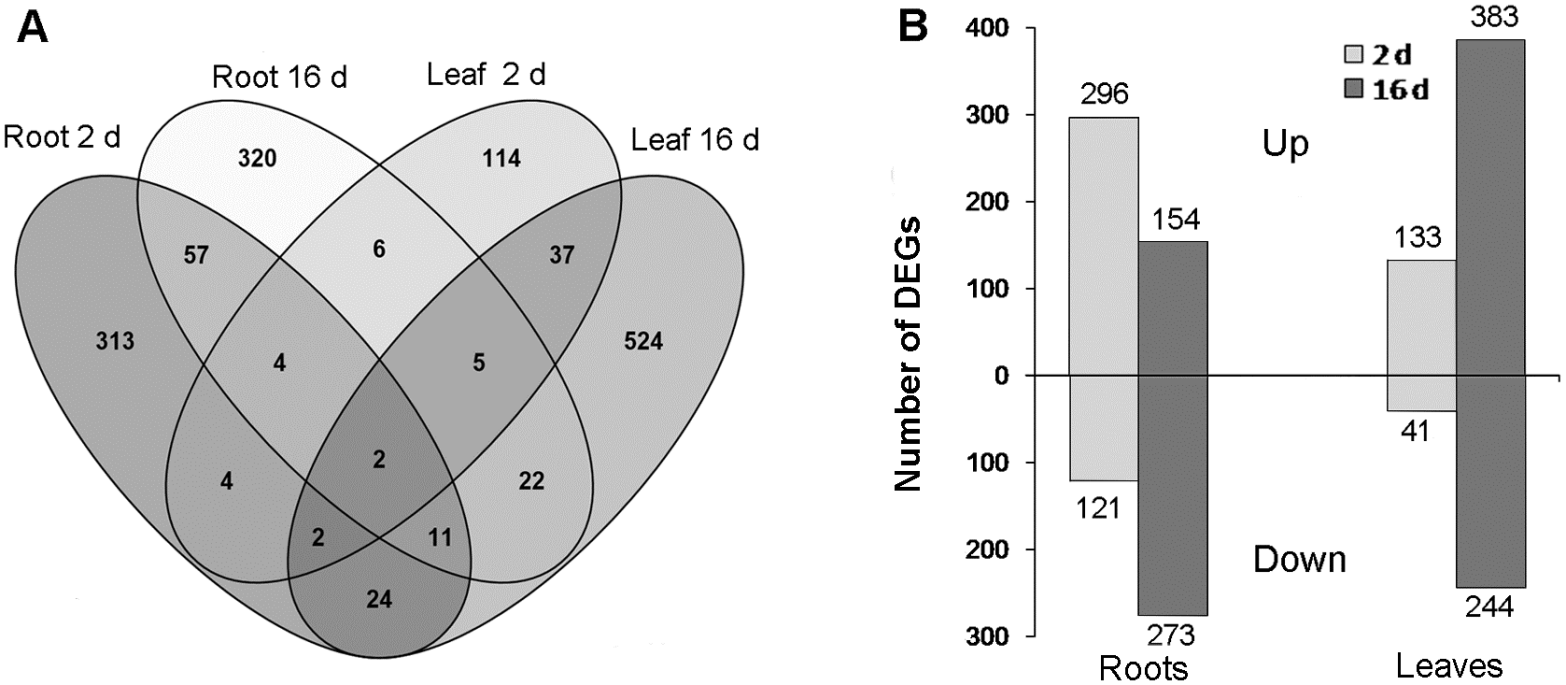
Statistics on DEGs responsive to N-deficiency in *J. Curcas*. A, Spatiotemporal distributions of DEGs; B, Distributions of up- and down-regulated genes.

N deficiency in *J. curcas* led to alternation in the expression of 1,445 DEGs, of which 770 were DEGs in roots and 755 were DEGs in leaves, 80 being common to both organs. In roots, 74 genes showed changes in expression at both 2 d and 16 d, while 343 or 353 genes were affected only at, respectively, 2 d or 16 d. In leaves, the expression of 46 genes was changed at both 2 d and 16 d, whereas 128 or 581 genes were affected, respectively, 2 d or 16 d alone. In roots, 313 and 320 genes showed changes in expression only at 2 d and only at 16 d respectively; in leaves, 114 and 524 genes were affected only at, respectively, 2 d and 16 d (Fig 1A and S2 Table). There were 296 and 154 genes that were activated, and 121 and 273 genes that were suppressed, respectively, after 2 d and 16 d of N removal in roots(Fig 1B). There were, respectively 133 and 383 up-regulated genes and 41 and 244 down-regulated genes at the same time points in leaves. Two DEGs were up-regulated at 2 d and 16 d in both organs, namely *JCGZ_16092* and *JCGZ_07030*, which were identified as *ASPARAGINASE B1* (*ASPGB1*) and *SARCOSINE OXIDASE* (*SOX*), respectively (S2 Table).

**Fig 2:**
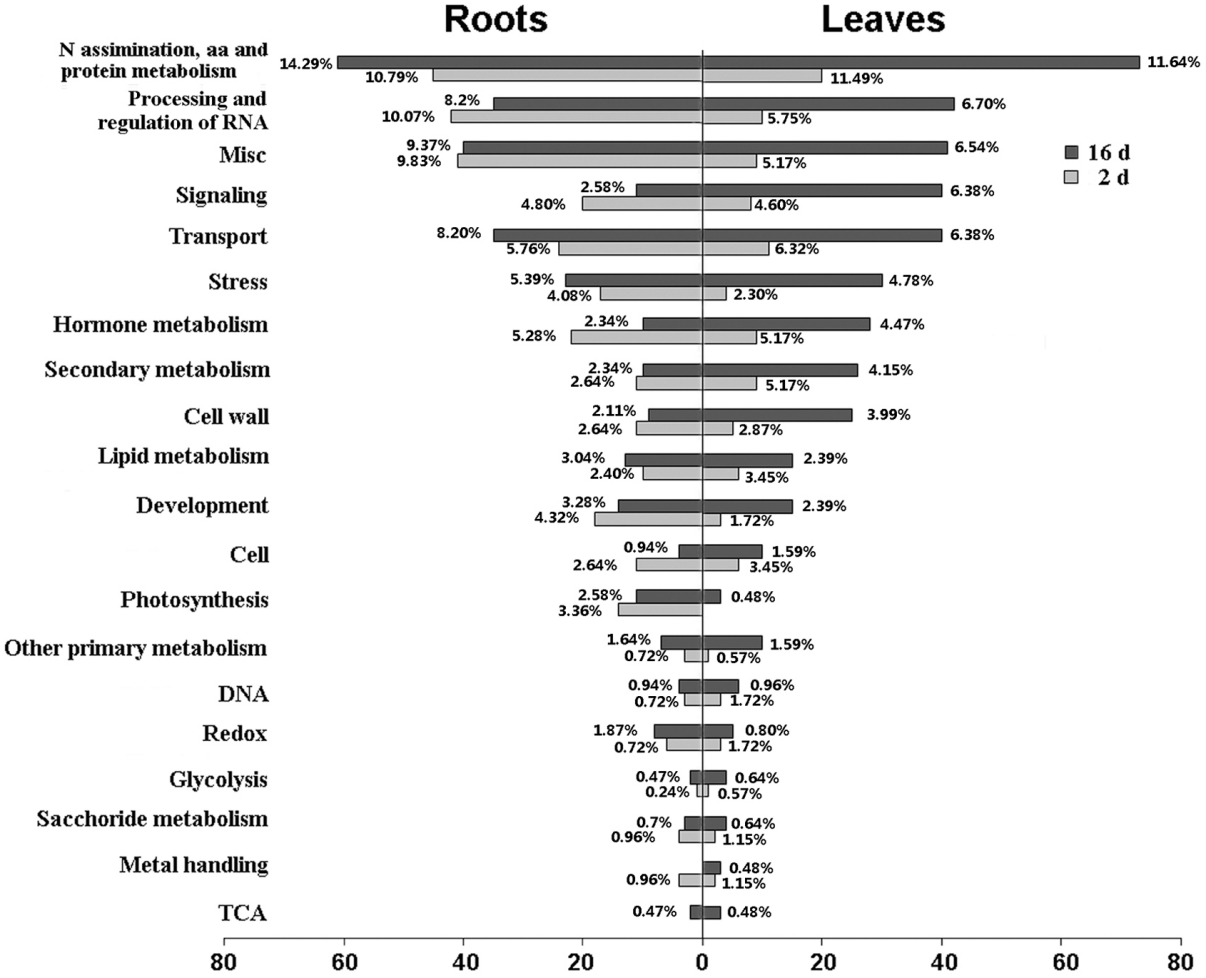
Classification of DEGs on the basis of MapMan clustering.

Classification of the DEGs revealed that N starvation influenced multiple biological processes(Fig 2). Of these processes, N assimilation, amino acid and protein metabolism pathways were the most strongly affected at 16 d in both roots and leaves. Genes in associated with processing and regulation of RNA, signaling and transport were also significantly enriched. Relatively speaking, the numbers of DEGs associated with C metabolism were fewer than those associated with N metabolism and secondary metabolism. In the following section, we discuss in more detail the organ-specificity of transcripts related to these particular pathways.

### DEGs in response to N starvation in roots

#### 1 Changes in expression of genes involved in N absorption, water and solute channels, and transport

**Fig 3:**
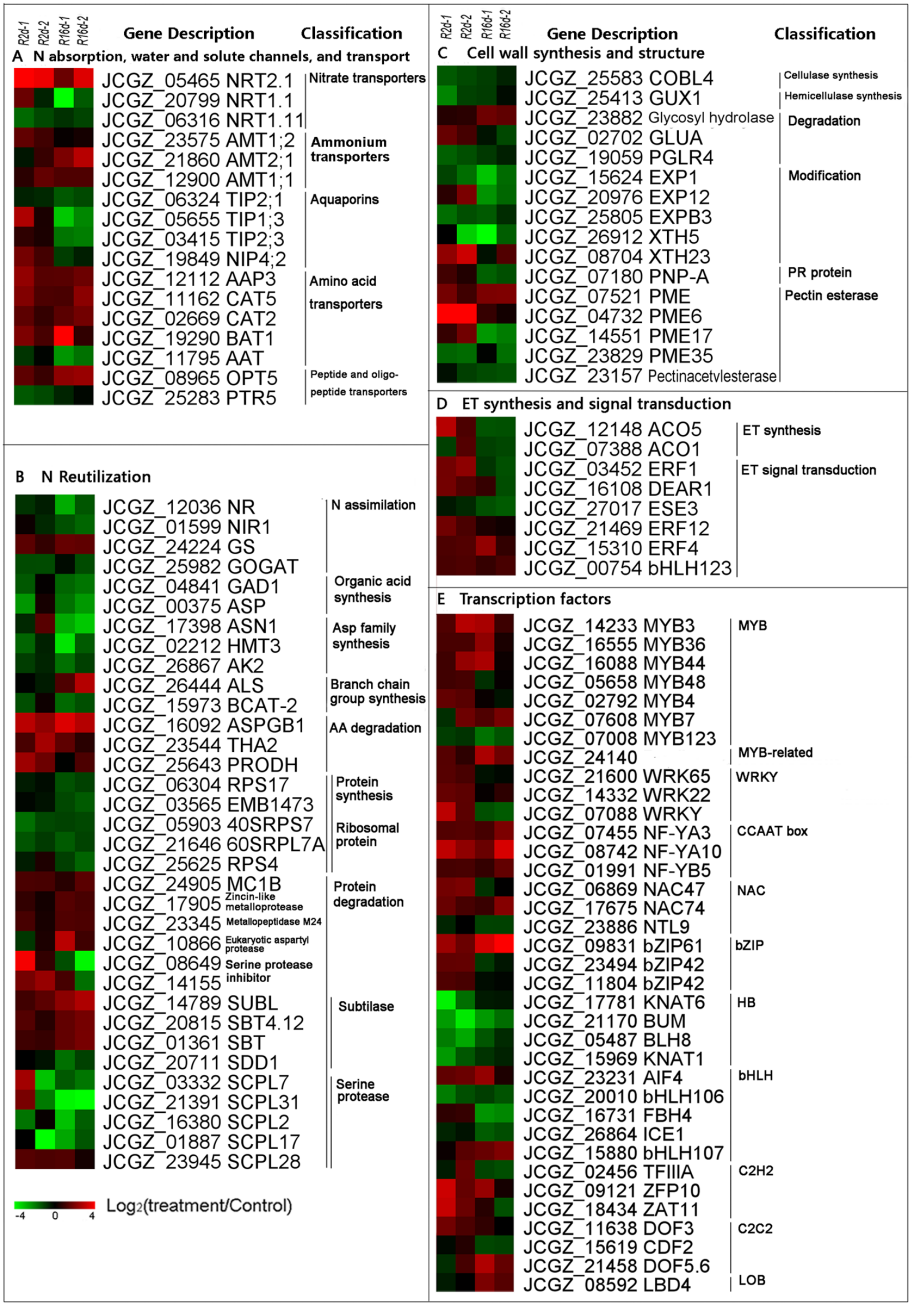
Classification of DEGs on the basis of MapMan clustering. A, N absorption, water and solute channels, and transport; B, N reutilization; C, Cell wall synthesis and structure; D, ethylene synthesis and signal transduction; E, transcription factors. R indicates root.

1. **N absorption.** Multiple genes involved in N absorption were differentially expressed under N starvation. Among the up-regulated sequences were *NRT2.1* and *AMTs*(Fig 3A and S3 Table). The expression levels of *JcNRT2.1* (*JCGZ_17788*) were up-regulated at 2 d and 16 d, suggesting that positive regulation of *NRT2.1* is maintained in *J. curcas* under long-term N deprivation. However, *JcNRT1.1* (*JCGZ_20799*) was repressed at 16 d, while the expression levels of *JcNRT1.11* (*JCGZ_06316*) were reduced at both time points. In the ammonium transporter family, the expression levels of *JcAMT1;1* (*JCGZ_12900*), *JcAMT1;2* (*JCGZ_23575*), and *JcAMT2;1* (*JCGZ_21860*) were increased at 2 d and/or 16 d of N-deficiency.
2. **Water and solute channels.** The genes encoding aquaporin (AQP) protein family members that are involved in N uptake showed altered expression during N deprivation (Fig 3A). Among these genes, three out of the four AQP homologs were significantly down-regulated at 16 d, namely *JcTIP2;1* (*JCGZ_06324*), *JcTIP1;3* (*JCGZ_05655*), and *JcTIP2;3* (*JCGZ_03415*), whereas only *JcNIP4;2* (*JCGZ_19849*) was up-regulated at 2 d and down-regulated at 16 d. The products of the first three of these genes are mainly localized to the vacuole membrane, while the last is on plasmalemma (S3 Table).
3. **Transport.** Genes with products involved in transporting organic nutrients, such as the AA transporter (AAT) gene families, were markedly up-expressed in response to N starvation, with *JcAAT* (*JCGZ_11795*) being an exception. Both *OLIGOPEPTIDE TRANSPORTER 5* (*JcOPT5*, *JCGZ_08965*) and *PEPTIDE TRANSPORTER 5* (*JcPTR5*, *JCGZ_25293*) belong to the peptide and oligopeptide transporter gene family. The former gene was significantly up-regulated at 16 d, whereas the latter was down-regulated at 2 d of stress. Other gene families associated with transport that were affected are list in S3 Table.

#### 2 DEGs associated with N reutilization

A shortage of N available for uptake severely affected genes associated with the conversion from inorganic to organic N complexes (Fig 3B and S4 Table). Among genes related to N assimilation process, the expression levels of *JcNR* (*JCGZ_12036*) and *JcNIR* (*JCGZ_01599*) were down-regulated at 16 d, whereas *JcGS* (*JCGZ_24224*) was up-regulated. The effects on these genes encoding key enzymes implied that N assimilation were attenuated under long-term N deficiency.

In AA metabolism, DEGs included those engaged in organic acid synthesis, aspartate family synthesis, branch chain group synthesis, and AA degradation. With respect to organic acid synthesis, the genes encoding *GLUTAMATE DECARBOXYLASE* (*GAD1*, *JCGZ_04841*) and *ASPARTATE AMINOTRANSFERASE* (*ASP*, *JCGZ_00375*) were both repressed at 16 d. The roles of the GAD1 and ASP proteins are to catalyze, respectively, the conversion of Glu to Gamma-aminobutyric acid and Asp to oxaloacetate. Genes encoding enzymes which are engaged in the synthesis of the aspartate family, including *JcASN1* (*JCGZ_17398*), *JcHMT3* (*JCGZ_02212*), and *JcAK2* (*JCGZ_26867*) were significantly inhibited after 16 d. In contrast, expression of those genes involved in AA degradation was enhanced; in particular, *JcASPGB1* (*JCGZ_16092*) was significantly up-regulated at both 2 d and 16 d. All reactions referred to here are detailed in S1 Fig.

With respect to protein metabolism, the DEGs were classified as falling into the protein synthesis and protein degradation category. In the protein synthesis group, the most strongly repressed genes were those of the family associated with ribosomal proteins, including 40S and 60S ribosomal protein structures. However, in the protein degradation category, different gene families showed differential patterns of altered expression. The expression levels of *METACASPASE 1B* (*JcMC1B*, *JCGZ_24905*), *ZINCIN−LIKE METALLOPROTEASE* (*JCGZ_17905*), and *METALLOPEPTIDASE M24* (*JCGZ_23345*) were increased. In Arabidopsis, *AtMC1* is a positive regulator of cell death [22]. The expression levels of genes involved in serine protease families were also markedly up-regulated, as exemplified by the activation of two *SUBTILASEs* (*JcSBT*, *JCGZ_20815* and *JCGZ_01361*) and one *SUBTILISIN−LIKESERINE PROTEASE* (*JcSUBL*, *JCGZ_14789*) after 16 d of N deprivation. In contrast, four out of five *SERINE CARBOXYPEPTIDES−LIKE* genes were strongly inhibited after 16 d.

#### 3 Changes in expression of genes associated with cell wall synthesis and structure

The DEGs in these category could be divided into the processes of cellulase and hemicellulase synthesis, degradation, modification, and pectin esterase(Fig 3C and S5 Table). Genes associated with cellulase and gemicellulase synthesis showed decrease in expression after 2 d. In the degradation category, the gene encoding *GLYCOSYL HYDROLASE* (*JCGZ_23882*) was greatly up-regulated after 16 d. Glycosyl hydrolase catalyzes the successive hydrolysis of *β*-D-glucose units from the non-reducing ends of 1,3-D-glucan, releasing *α*-glucose. In the modification group, three *EXPANSINs* (*EXPs*, *JCGZ_15624*, *JCGZ_20976*, and *JCGZ_25805*) were significantly repressed after 16 d. In the pectin esterase class, the expression levels of four *PECTIN METHYLESTERASE* (*PME*) genes were changed at 2 d and/or 16 d. In particular, *PME54* (*JCGZ_07521*) was up-regulated at both 2 d and 16 d. The enzyme PME hydrolyzes pectin to release pectate and methanol.

#### 4 DEGs involved in phytohormone metabolism and TF families

In roots, the expression patterns of genes related to ethylene (ET) synthesis were affected in opposite directions by 2 d and 16 d stresses (Fig 3D). For instance, the expression of *JCGZ_12148* encoding *AMINOCYCLOPROPANE CARBOXYLATE OXIDASE 5* (*ACO5*) increased at least 2.3-fold at 2 d, while it decreased by, on average, 2.4-fold after 16 d, at which point *JCGZ_07388* (*ACO1*) was also down-regulated. The transcription levels of the downstream gene *ETHYLENE RESPONSE FACTOR 1* (*ERF1*) showed the same pattern of rise and fall, which was also displayed by *ALLENE OXIDE SYNTHASE* (*AOS*, *JCGZ_10035*) and *JASMONATE O−METHYLTRANSFERASE* (*JMT*, *JCGZ_22158*), which both participate in jasmonate (JA) synthesis (S6 Table).

Under N starvation, the TFs affected included MYB, MYB-related, CCAAT box, NAC, bZIP, HB, bHLH, C2H2, C2C2, and LOB gene families(Fig 3E and S7 Table). The genes in the MYB, WRKY, NAC and bZIP families were up-regulated mainly at 2 d, whereas transcripts of HB family members were suppressed at that time point. Three of CCAAT box genes, *JCGZ_07455*, *JCGZ_08742*, and *JCGZ_01991*, were activated under 2 d and 16 d starvation; they encode, respectively, *JcNAC47*, *JcNAC74*, and *NAC TRANSCRIPTION FACTOR−LIKE 9* (*JcNTL9*). In addition, *JcLBD4* (*JCGZ_08592*) in the LOB family was activated after long-term N deficiency.

### DEGs in response to N starvation in leaves

#### 1 Changes in expression of genes involved in N absorption and transport

**Fig 4:**
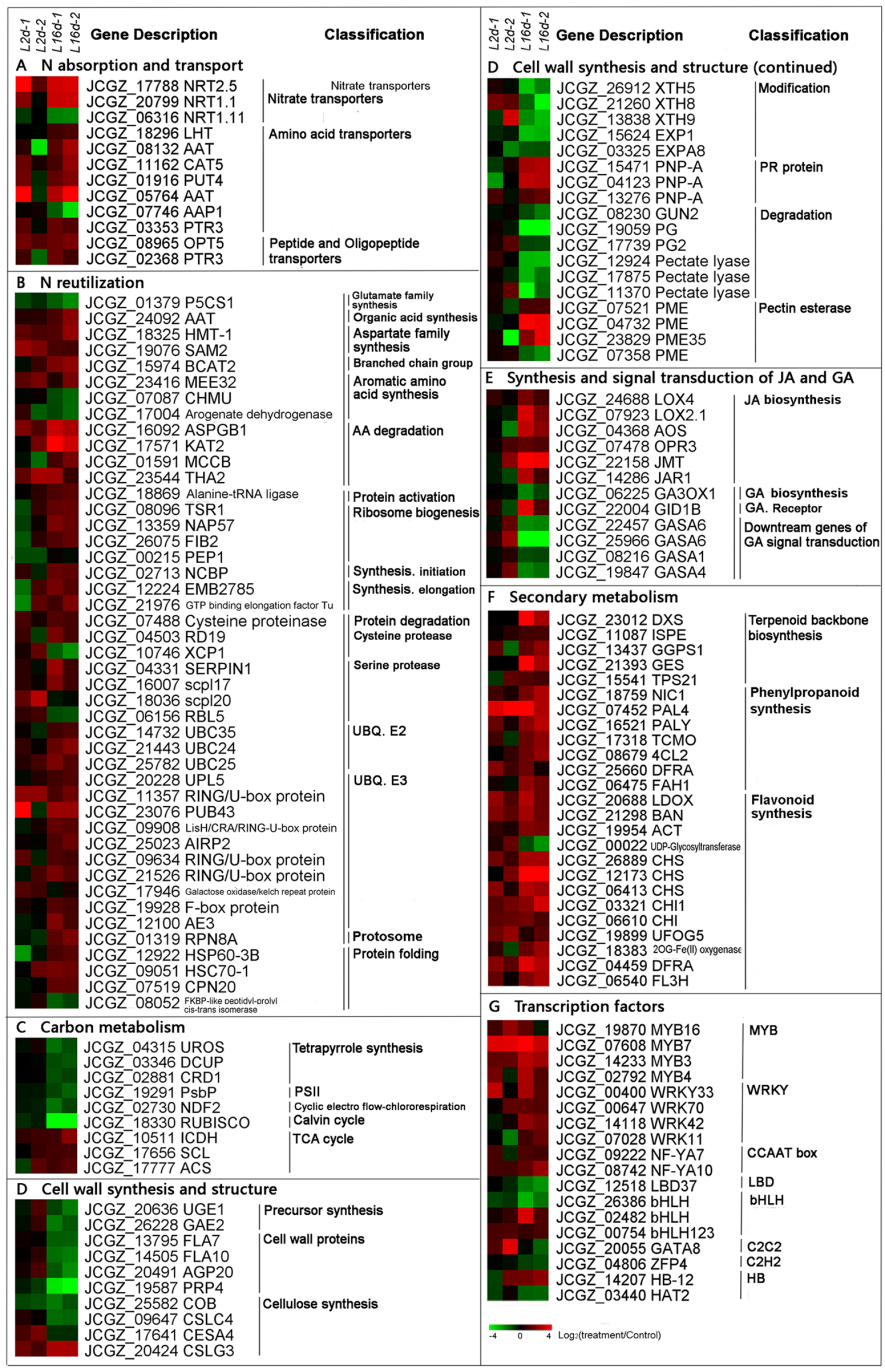
Heatmaps showing DEGs in leaves after 2 d and 16 d of N starvation (two biological replicates in each case). A, N absorption and transport; B, N reutilization; C, carbon metabolism; D, cell wall synthesis and structure; E, synthesis and signal transduction of JA and GA; F, secondary metabolism; G, transcription factors. L indicates leaves.

1. **N absorption.** In leaves, the DEGs involved in nitrogen transport mainly encompassed NRTs, AATs, and peptide and oligopeptide transporters (Fig 4A and S8 Table). In the NRT gene family, the expression levels of *JcNRT2.5* (*JCGZ_17788*) and *JcNRT1.1* (*JCGZ_20799*) were up-regulated more than 9-fold after 16 d, while *NRT1.11* was approximately repressed approximately 4-fold.
2. **Transport.** A total of seven DEGs were assigned to the AAT gene family of amino acid transporters. Six out of these seven genes were significantly up-regulated after 16 d. The types of AAT family genes that showed altered expression were diverse, including *LYSINE/HISTIDINE TRANSPORTER* (*JCGZ_18296*), *CATIONIC AMINO ACID TRANSPORTER 5* (*CAT5*, *JCGZ_11162*), *POLYAMINE UPTAKE TRANSPORTER* (*PUT4*, *JCGZ_01916*), *AAT* (*JCGZ_08132* and *JCGZ_05764*), and *AMINO ACID PERMEASE 1* (*AAP1*, *JCGZ_07746*). With the exception of *JCGZ_07746* (*AAP1*), which was down-regulated after 16 d, these genes were all significantly up-regulated at that time point. Three genes in peptide and oligopeptide transporters category were also activated, namely two *PTR3s* (*JCGZ_03353* and *JCGZ_02368*) and *OPT5* (*JCGZ_08965*). Both *PTR3s* showed increased expression at 16 d, while *OPT5* was up-regulated after both medium- and long-term N starvation.

#### 2 N reutilization

N reutilization encompasses AA and protein metabolism(Fig 4B and S9 Table). The DEGs related to N primary metabolism could be divided into AA synthesis and degradation. Unlike the situation in roots, there were no significantly changes in expression of genes associated with N assimilation in leaves. The DEGs participating in organic N synthesis were subdivided into the categories glutamate family synthesis, organic acid synthesis, aspartate family synthesis, branched chain group and aromatic AA synthesis. Details of the reactions in these metabolic pathways were listed are given in S2 Fig. Except for *PYRROLINE−5−CARBOXYLATE SYNTHASE 1* (*P5C51*, *JCGZ_01379*), a member of the glutamate family synthesis, which was inhibited at 16 d, the genes related to AA synthesis processes were activated under medium- and/or long-term starvation.

In the AA degradation process category, four transcripts showed raised levels of expression, these were *ASPGB1* (*JCGZ_16092*), *3−KETOACYL−CoA THIOLASE* (*KAT2*, *JCGZ_17571*), *METHYLCROTONYL−CoA* (*MCCB*, *JCGZ_01591*), and *1−ALLO−THREONINE ALDOLASE* (*THA2*, *JCGZ_23544*). As mentioned above, *JcASPGB1* (*JCGZ_16092*), which participates in AA degradation, was strongly up-regulated not only in leaves but also in roots.

In the protein metabolism category, the DEGs were divided into protein synthesis, protein degradation and protein folding. In the protein synthesis, seven out of eight genes, which were distributed among the protein activation, ribosome biogenesis, initiation, and elongation processes, were up-regulated mainly after 16 d of N starvation. In the protein degradation category, expression of most of the genes encoding members of the cysteine protease, serine protease and ubiquitin (UBQ) families was enhanced, in particular, 13 *UBQ* genes were up-regulated. In the protein folding group, three out of the four genes were up-regulated.

#### 3 DEGs associated with carbon (C) metabolism

Expression of several genes associated with C assimilation was significantly repressed under 16 d of N deprivation; these genes included *UROPORPHYRINOGEN III SYNTHASE* (*UROS*, *JCGZ_04315*), *UROPORPHYRINOGEN DECARBOXYLASE* (*DCUP*, *JCGZ_03346*), and *MAGNESIUM−PROTOPORPHYRIN IX MONOMETHYL ESTER CYCLASE* (*CRD1*, *JCGZ_02881*), all of which participate in the synthesis of the chlorophyll precursor tetrapyrrole; *PsbN* in PSII, *NDH−DEPENDENT CYCLIC ELECTRON FLOW 1* (*NDF2*, *JCGZ_02730*) in the cyclic electron flow-chlororespiration system, and *RUBISCO* (*JCGZ_19291*) in the Calvin cycle (Fig 4C and S10 Table). In contrast, three DEGs involved in the TCA cycle showed significant increases in expression, *ISOCITRATE DEHYDROGENASE C* (*ICDH*, *JCGZ_10511*), *SUCCINYL−CoA LIGASE* (*SCL*, *JCGZ_17656*) and *ATP−CITRATE SYNTHASE*(*ACS*, *JCGZ_17777*)(S3 Fig).

#### 4 DEGs involved in cell wall synthesis and structure

The genes related to the cell wall that showed altered expression in leaves under long-term N starvation included some involved in precursor synthesis, cell wall proteins, cellulose synthesis, modification, degradation, and pectinesterase (Fig 4D and S11 Table). Some of reactions in these pathways are listed in S4 Fig. Among these processes, transcripts in precursor synthesis, cell wall proteins and cellulose synthesis were obviously repressed. In precursor synthesis, *JCGZ_20636* and *JCGZ_26228* encode UDP-GLUCOSE 4-EPIMERASE(UGE1) and UDP-D-GLUCURONATE 4-EPIMERASE 2 (GAE2), respectively. Expression levels of both *UGE1* and *GAE2* were down-regulated after 16 d. Enzyme UGE1 catalyzes the reversible transformation of UDP-*α*-glucose to UDP-*α*-galactose, while UGE1 carried out the interconversion of UDP-*α*-glucuronate and UDP-*α*-galacturonate(S4 Fig reaction 28-29). *JcFLA7* (*JCGZ_13795*), *JcFLA10* (*JCGZ_14505*), and *JcAGP20* (*JCGZ_20491*) which belong to the *ARABINOGALACTAN* (*AGP*) gene family, were strongly repressed after 16 d starvation. In the modification subgroup, intriguingly, three genes encoding XYLOGLUCAN ENDOTRANSGLUCOSYLASE/HYDROLASE (XTHs) and two *EXPs* were significantly repressed. Expression levels of three genes encoding PLANT NATRIURETIC PEPTIDE A (PNP-A) were increased; these encode a newly identified pathogenesis related (PR) protein with chitinase activity. Those genes involved in degradation process, such as *ENDOGLUCANASE* (*GUN*, *JCGZ_08230*), *POLYGALACUTURANASE* (*PGs*, *JCGZ_19059* and *JCGZ_17739*) and three *PECTIN LYASEs*, were strongly repressed. In contrast, the expression levels of three *PMEs* (*JCGZ_07521*, *JCGZ_04732* and *JCGZ_23829*) were increased.

#### 5 Changes in expression of genes related to secondary metabolism

Genes associated with terpenoid backbone biosynthesis, and the synthesis of phenylpropanoids and flavonoids were initially up-regulated at 2 d and their expression was conspicuously enhanced after 16 d treatment (Fig 4F and S12 Table). It is worth emphasizing that genes encoding key enzymes in the synthesis of secondary metabolites were identified, including two *JcPALs* (*JCGZ_07452* and *JCGZ_16521*), three *JcCHSs* (*JCGZ_26889*, *JCGZ_12173*, and *JCGZ_06413*), and two *JcCHIs* (*JCGZ_03321* and *JCGZ_06610*) (S6 Fig).

#### 6 DEGs involved in phytohormone metabolism and transcription factor gene families

In phytohormone pathways, DEGs associated with JA synthesis were all significantly up-regulated(Fig 4G and S13 Table); they included *LOX2* (*JCGZ_07923*), *LOX4* (*JCGZ_24688*), *AOS* (*JCGZ_04638*), *OPR3* (*JCGZ_07478*), and *JMT* (*JCGZ_22158*) (S5 Fig). Additionally, the expression of *JAR* (*JCGZ_14286*) was increased; the enzyme JAR conjugates JA and isoleucine. However, the expression of *GA3 β−HYDROXYLASE 1* (*GA3OX1*, *JCGZ_06225*), encoding an enzyme of gibberelin (GA) synthesis, was inhibited and the negative receptor gene *GA INSENSITIVE DWARF 1B* (*GID1B*) was up-regulated. The following genes *GASA6s* (*JCGZ_22457* and *JCGZ_22457*), *GASA1* (*JCGZ_08216*), and *GASA4* (*JCGZ_19847*), which encode proteins downstream of the receptor in the GA signaling cascade, were also down-regulated at 16 d.

In leaves, the TFs that showed changes in expression fell into the MYB, WRKY, CCAAT box, LOB, bHLH, C2H2, C2C2, and HB gene families(Fig 4G and S14 Table). The DEGs in the MYB, WRKY, and CCAAT families were up-regulated mainly at 16 d. It should be noted that *JcLBD37* was down-regulated in leaves, while its homolog *JcLBD4* was up-regulated in roots.

### RT-qPCR verification

**Fig 5:**
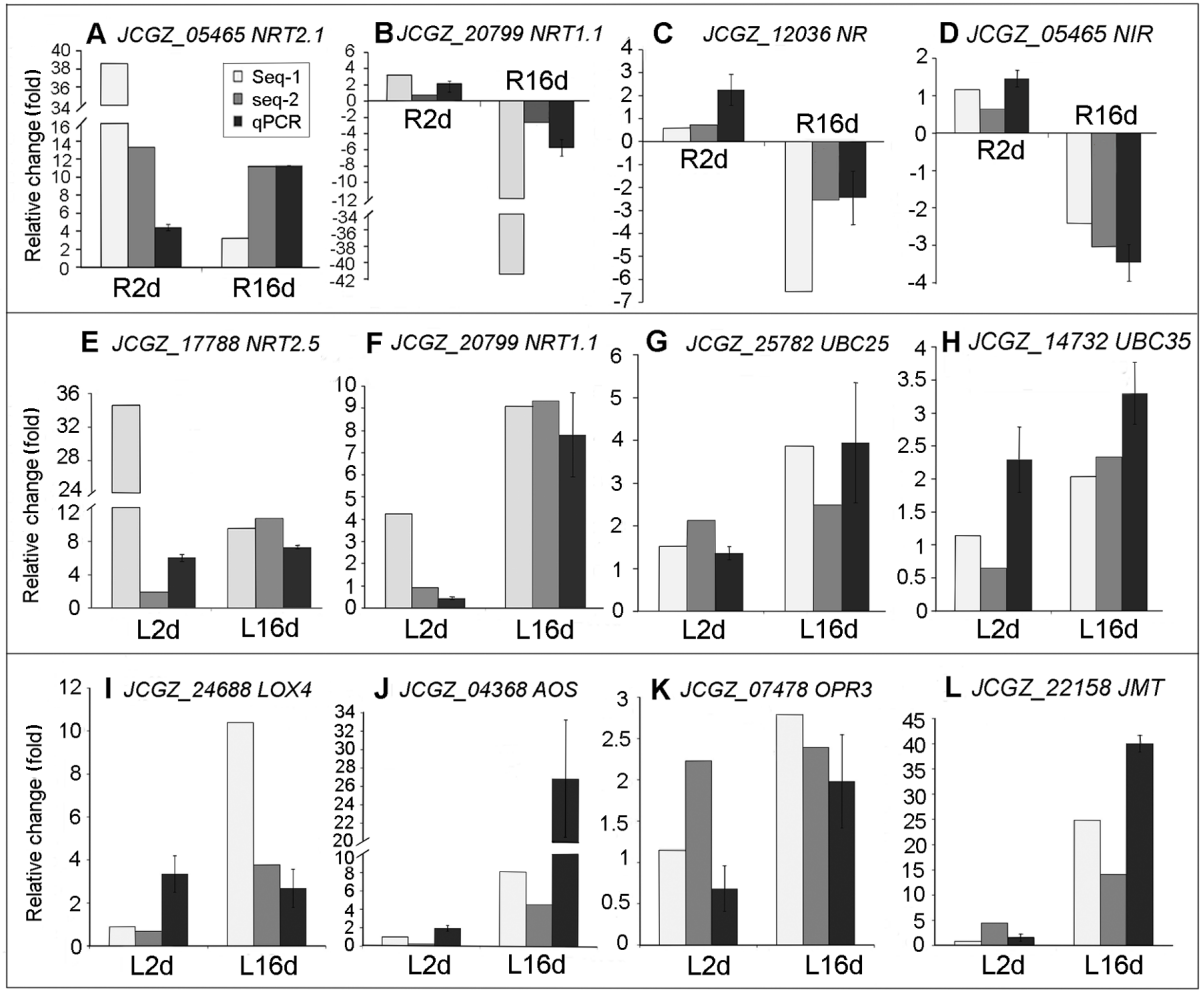
Relative changes in gene expression measured by RT-qPCR. A–D: *NRT2.1*, *NRT1.1*, *NR*, and *NIR* in roots; E–H: *NRT2.5*, *NRT1.1*, *UBC25*, and *UBC35* in leaves; I–L: *LOX4*, *AOS*, *OPR3*, *JMT*, components of the jasmonate synthesis pathway, in leaves.

Genes included in this analysis were *NRT2.1* (*JCGZ_05465*), *NRT1.1* (*JCGZ_20799*), *NR* (*JCGZ_12036*), and *NIR* (*JCGZ_01599*) in roots, *NRT2.5* (*JCGZ_17788*), *NRT1.1* (*JCGZ_20799*), *UBC25* (*JCGZ_25782*), and *UBC35* (*JCGZ_14732*) in leaves, and the genes *LOX4* (*JCGZ_24688*), *AOS* (*JCGZ_04638*), *OPR3* (*JCGZ_07478*), and *JMT* (*JCGZ_22158*) (a component of the JA synthesis pathway in leaves) which had shown striking alterations in expression (Fig 5). Taking into account the differences in environmental conditions in different years, the patterns revealed by the results of the two techniques were quite consistent.

## Discussion

By analyzing the profiles obtained from RNA-seq technology, we successfully identified a set of gene modules whose expression was altered in physic nut plants under N limitation. MapMan classification showed that DEGs associated with the processes of N assimilation and AA and protein metabolism were predominated under stress in both roots and leaves (Fig 2). DEGs with roles of processing and regulation of RNA, signaling and transport were also significantly enriched. Because transporters are the crucial gateways through which nutrient elements enter plants, the functions of N transporters and the genes that encode them have been intensively studied. Meta-analysis has shown that the ion transport gene network module is robustly responsive to nitrate in Arabidopsis roots [10]. Among the transporters, members of high-affinity NRT2 families play important roles in N uptake under low N conditions [23]. In our profiling study, the levels of *JcNRT2.1* transcript in roots were up-regulated at both 2 d and 16 d of N deprivation, while both *JcNRT1.1* and *JcNRT1.11* were repressed after 16 d of stress (Fig 3A). In leaves, however, the up-regulated NRT genes were *JcNRT2.5* and *JcNRT1.1* (Fig 4A). The dual-affinity Arabidopsis protein AtNRT1.1 has receptor activities which can operate over a wide range of concentrations [24]. Under conditions of severe N starvation, AtNRT2.5 contributes to the phloem loading of NO_3_^−^ in Arabidopsis shoots and functions with AtNRT2.1, AtNRT2.2, and AtNRT2.4 to support shoot growth [25]. We inferred that the high-affinity N transporters play leading roles under N limitation in roots, while the coordinated action of JcNRT1 and JcNRT2 makes leaves more adaptable to variations in N concentration. In addition, *JcAMTs* were up-regulated, probably to promote the uptake of ammonia under stress (Fig 3A). Most membrane channel AQP proteins facilitate efficient transfer of water and small neutral molecules across biological membranes, and they are extremely sensitive to nitrogen deprivation in plants [26]. In our study, the expression of *JcTIP2;1*, *JcTIP1;3*, and *JcTIP2;3* was reduced in roots, suggesting that in these plants internal osmotic stress was increased by a reduction in the levels of expression of *AQPs* (Fig 3A).

Nitrogen uptake is coupled with N assimilation and the remobilization of organic nitrogen at the whole-plant level [27]. Under N deprivation, *JcNR*, *JcNIR*, *JcGOGAT*, and genes related to the synthesis of organic acids and Asp family AAs were negatively regulated in roots, suggesting that not only N assimilation but also the production of AAs was reduced (Fig 3B). In Arabidopsis, organic N induces *AtNR* and *AtASN1*, but negatively regulates *AtAMT1.1* [28]. Thus, the inhibition of *JcASN1* expression and the up-regulation of *JcAMT1.1* are consistent with the reduction in N availability (Fig 3A and 3B). In leaves, many genes associated with AA synthesis and degradation and protein degradation were up-regulated (Fig 4B). In particular, the gene *ASPGB1* was significantly up-regulated in both roots and leaves (S2 Table). Under long-term exposure of reproductive-stage wheat to low N, *ASPG* was also significantly up-regulated in stems and leaves [9]. Expression of genes in the ubiquitination pathway for protein degradation, including those encoding enzymes of the E2 and E3 families, was increased (Fig 4B). Long-term N limitation induced the same effect in Arabidopsis. For instance, ubiquitin ligase *nla* mutants grown with low N failed to degrade N metabolites [29]. Moreover, the genes encoding Rubisco and enzymes required for the synthesis of chlorophyll precursors were dramatically repressed in our studies, indicating that N-containing reserves were being remobilized (Fig 4C). Rubisco is the main leaf N storage protein, typically representing 30-50% of the total protein [30], [31]. Expression of genes in the AAT family was strongly activated in both leaves and roots (Figs 3A and 4A). Amino acids distributed by AATs after the N assimilation and reduction of inorganic N root or shoot tissue [32]. Because AAs have a wide range of structures and physical properties, most plants possess multiple AATs. For example, AtBAT1 is a bi-directional transporter which is a possible exporter for certain AAs [33], while AtCAT5 prefer basic AAs, and AtCAT3, AtCAT6 and AtCAT8 prefer neutral or acidic AAs [34]. Among the peptide and oligopeptide transporter genes, *JcPTR*, *JcOPT5*, and *JcPTR3* showed increased expression in leaves. In Arabidopsis, AtPTR proteins carry di- and tri-peptides, while AtOPTs transport tetra- and penta-peptides, and glutathione [32]. Our data thus suggest that organic N transporter activities increased under conditions of N deficiency, leading to an increase in the degradation of organic N-containing compounds and export of the resulting products from source organs (leaves) to sink organs in order to enable the latter to deal with the consequences of N starvation.

The interconnections between C fixation and N metabolism are complex. C skeleton and substantial amount of energy needed for N reduction derive from photosynthesis, photorespiration and respiration [35]. In Arabidopsis, concentrations of sugars and starch increased or remained unchanged under N deficiency [7], [11], [31]. We observed that few genes encoding PSII component underwent changes in expression in leaves during mid- or long-term N limitation (Fig 4C). On the other hand, the degradation of AAs and proteins release large quantities of C skeleton that can cause a C/N imbalance. A high C/N ratio can induce anthocyanin accumulation, which is one of the traits typically observed under N deprivation [36], [37], [38]. In our study, genes associated with the biosynthesis of terpenoid back-bones, phenylpropanoids, and flavonoids were dramatically up-regulated after 16 d; these genes included three *JcCHSs* and two *JcCHIs* (Fig 4F). The phytohormone JA can play pivotal roles in C and secondary metabolism. In our profiling analysis, six genes encoding component of the JA biosynthesis pathway were strongly up-regulated under N deprivation (Fig 4E). JA not only works as an elicitor to increase the accumulation of secondary metabolites [39, 40], but also induces AA reallocation from leaves towards roots [41]. In reproductive stage wheat, *AOC* and *OPR*, which encode enzymes of JA biosynthesis, were expressed at higher levels in all organs under low N [9]. We therefore suppose that increased JA signaling led to anthocyanin accumulation, chlorophyll degradation and acceleration of AA reallocation. JA signaling can recruit some members of the NAC, bHLH, R2R3-MYB, and WRKY families [42]. In our profiles, the genes for several TFs in the MYB and WRKY families were up-regulated after 16 d of nitrogen starvation, showing patterns similar to those of genes related to secondary metabolism and AATs. In Arabidopsis, some MYBs controlled the accumulation of anthocyanins and flavonoids in leaves under conditions of N depletion [43], [44], [45], and regulated the expression of N assimilation genes, such as *AtGS* and *AtASN* [46], [47]. *AtLBD 37/38/39* genes negatively regulated anthocyanin biosynthesis, N uptake and assimilation [48], [49]. We found that JcLBD37 was down-regulated at 16 d in leaves (Fig 4G).

There is no doubt that N limitation has a major effect on cell structure. Ribosomes play regulatory roles in development [50]. Expression of genes associated with protein synthesis and of RP genes was reduced under N stress (Fig 3B), possibly leading to inadequate biosynthesis of protein components including certain enzymes in roots. EXPs, PNP, and XTHs are types of cell wall protein [51], [52], [53]. Expression of *JcEXPs* and *JcXTHs* in both leaves and roots was greatly reduced after 16 d (Fig 3C), whereas expression of *JcPNAs* in leaves increased at the same time point (Fig 4D). GA signaling was inhibited by the down-regulated of *GA3OX1* and four *GASAs* that interact with the negative receptor GID1B were up-regulated in leaves. *GASA* genes are implicated in cellular processes such as cell division and expansion [54]. These results suggested that cellular development in both organs was repressed under N deficit. ET inhibits primary root elongation, and ERF1 can stimulate auxin accumulation and ethylene-induced inhibition of root growth [55], [56]. ERF1 is also a downstream component in both ET and JA signaling and is involved in pathogen resistance [55]. *JcACO5* and *JcACO1*, which encode enzymes of in ET synthesis, and *JcETR1*, showed increased expression at 2 d but their expression was repressed at 16 d (Fig 3D), suggesting that primary root elongation was released from inhibition by ET signaling under long-term N-starvation, with a concomitant reduction in pathogen resistance. Further investigation into the potential role of N deficiency-induced cell wall modifications may shed light upon host-pathogen-phytohormone interactions. Other TFs also play important extensive roles in cell structure. *PtaLBD4* and *PtaLBD1* are specifically up-regulated in secondary phloem in poplar [57]. We found that *JcLBD4* was increased after 16 d in roots. *NACs* are involved in developmental programs, senescence, formation of secondary walls, and biotic and abiotic stress responses [58]. *JcNAC74* increased at both 2 d and 16 d (Fig 3E). Long-term N starvation promotes the expressions of *AtNF−YA2, 3, 5, 7*, and *10* in Arabidopsis, and plants over-expressing *AtNF−YA2/7/10* have a dwarf phenotype caused by the impairment of cell elongation and they accumulate higher starch content [59]. *JcNF−YA3, 5*, and *10* were up-regulated mainly in roots, and *JcNF−YA7, 10* transcripts increased in leaves in response to N deficiency (Figs 3E and 4G). The activation of *JcNF−YA10* could be associated with abnormal growth under N deficiency. Proteins of the WRKY family are considered to modulate plant development and respond abiotic stresses [60]. In leaves, four *JcWRKYs* were induced under long-term N starvation, possibly induced by JA and they may offset the negative effect of *ERF1*.

## Conclusion

**Fig 6:**
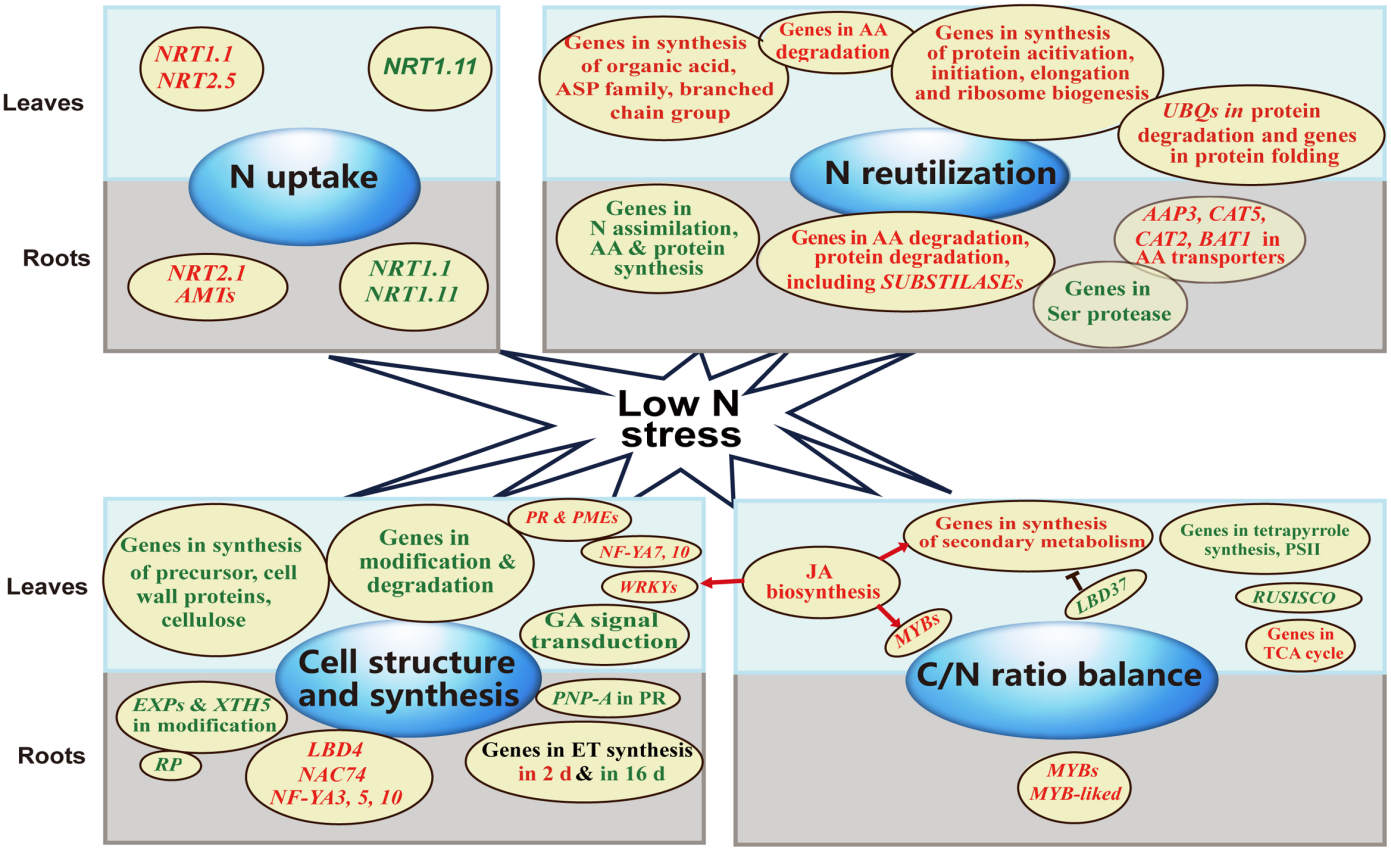
Preliminary schematic map of DEGs in physiological processes responding to N starvation in *J. curcas* roots and leaves. Red represents up-regulation, dark green indicates down-regulation.

By analyzing transcriptional profile, we showed that four major groups of genes were influenced by long-term N limitation organ-specific, including those encoding components controlling N uptake, N reutilization, C/N ratio balance, and cell structure and synthesis. We outline the regulatory framework (Fig 6). Our discoveries may offer clues to the molecular mechanisms that regulate N reallocation and reutilization in order to maintain or increase plant performance even under adverse environmental conditions.

## Supporting information

S1 Fig Enzymes encoded by differentially expressed genes involved in N assimilation and amino acid metabolic pathways in roots. The red arrow represents increase gene expression; the green arrow represents decreased expression.
(TIF)

S2 Fig Enzymes encoded by differentially expressed genes involved in amino acid metabolic pathways in leaves. (TIF)

S3 Fig Enzymes encoded by differentially expressed genes involved in the TCA cycle in leaves. (TIF)

S4 Fig Enzymes encoded by differentially expressed genes involved in cell wall structure in leaves. (TIF)

S5 Fig Enzymes encoded by differentially expressed genes involved in jasmonate synthesis in leaves. (TIF)

S6 Fig Key enzymes encoded by differentially expressed genes involved in secondary metabolism in leaves. (TIF)

S1 Table Sequences of primers used for RT-qPCR. (XLSX)

S2 Table DEGs identified in roots and/or leaves after 2 d and/or 16 d of N deficiency. Values are gived as log2(TPMtreatment/ TPMcontrol), R, root; L, leaves.
(XLSX)

S3 Table DEGs involved in N absorption, water and solute channels, and transport in roots. (XLSX)

S4 Table DEGs related to in N reutilization in roots. (XLSX)

S5 Table DEGs associated with cell wall synthesis and structure in roots. (XLSX)

S6 Table DEGs associated with phytohormone biosynthesis and signaling transduction in roots. (XLSX)

S7 Table DEGs associated with transcription factor families in roots. (XLSX)

S8 Table DEGs involved in N absorption, water and solute channel, and transport in leaves. (XLSX)

S9 Table DEGs related to N reutilization in leaves. (XLSX)

S10 Table DEGs involved in carbon (C) metabolism in leaves. (XLSX)

S11 Table DEGs associated with cell wall synthesis and structure in leaves. (XLSX)

S12 Table DEGs associated with phytohormone metabolism and signaling transduction in leaves. (XLSX)

S13 Table DEGs associated with secondary metabolism in leaves. (XLSX)

S14 Table DEGs associated with transcription factor families in leaves. (XLSX)
