# Supplementary figures and images for: Global gene expression analysis of the response of physic nut (Jatropha curcas L.) to medium- and long-term nitrogen deficiency
Source: PLoS One. 2017 Aug 17;12(8):e0182700. doi: 10.1371/journal.pone.0182700 (PMC5560629; doi:10.1371/journal.pone.0182700)

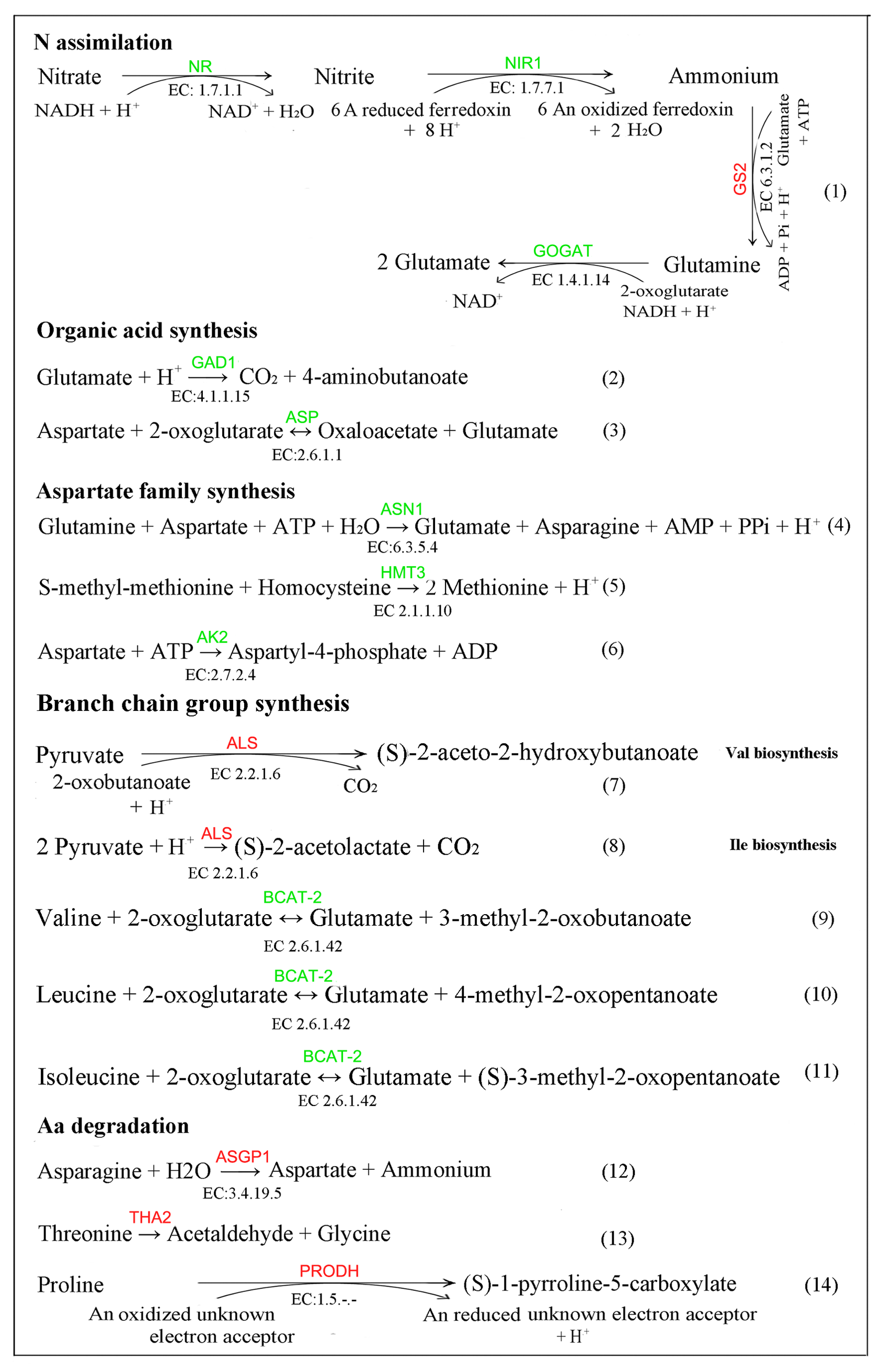

Supplement: S1 Fig — The red arrow represents increase gene expression; the green arrow represents decreased expression. (TIF) [file pone.0182700.s001.tif]

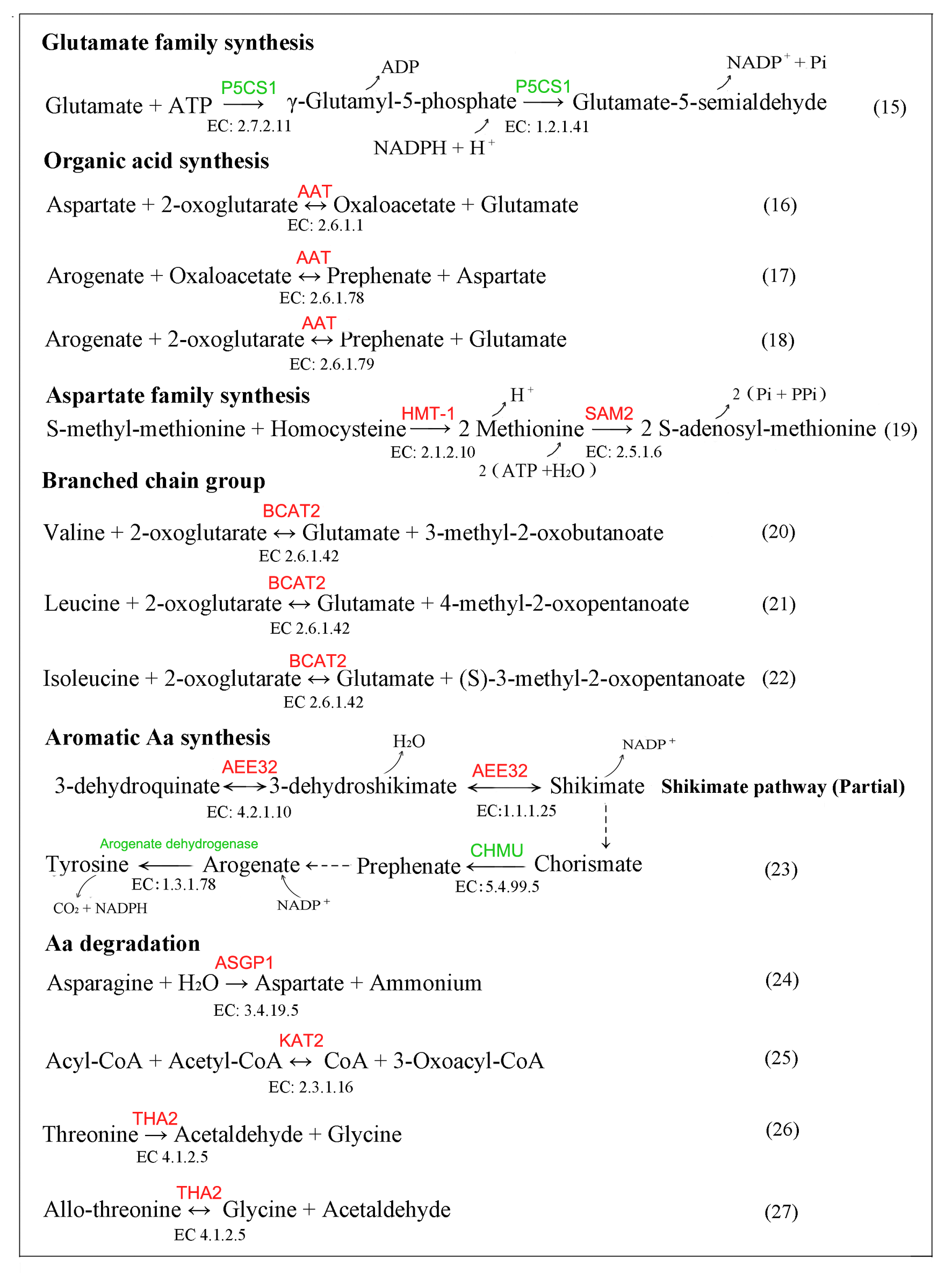

Supplement: S2 Fig — (TIF) [file pone.0182700.s002.tif]

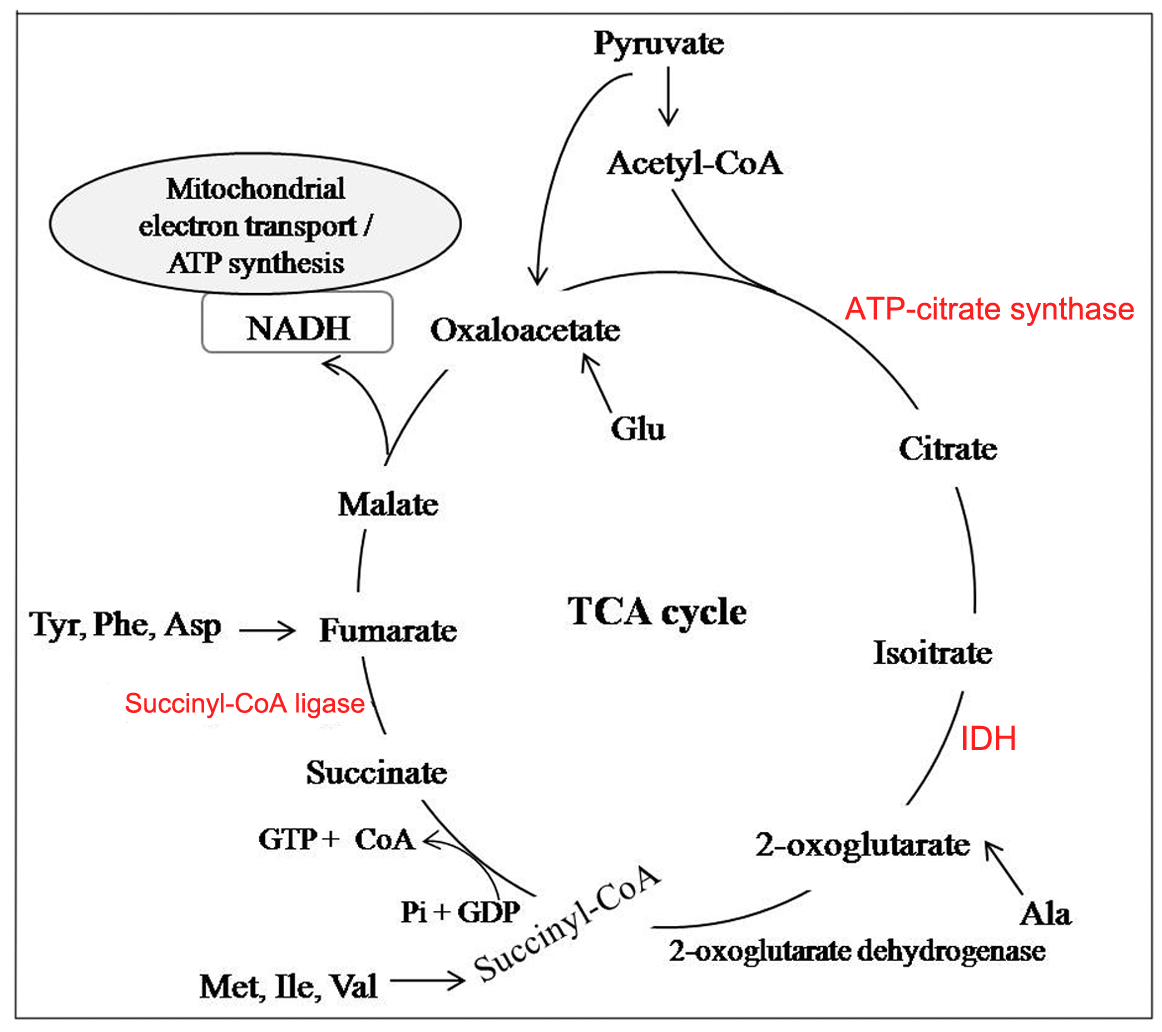

Supplement: S3 Fig — (TIF) [file pone.0182700.s003.tif]

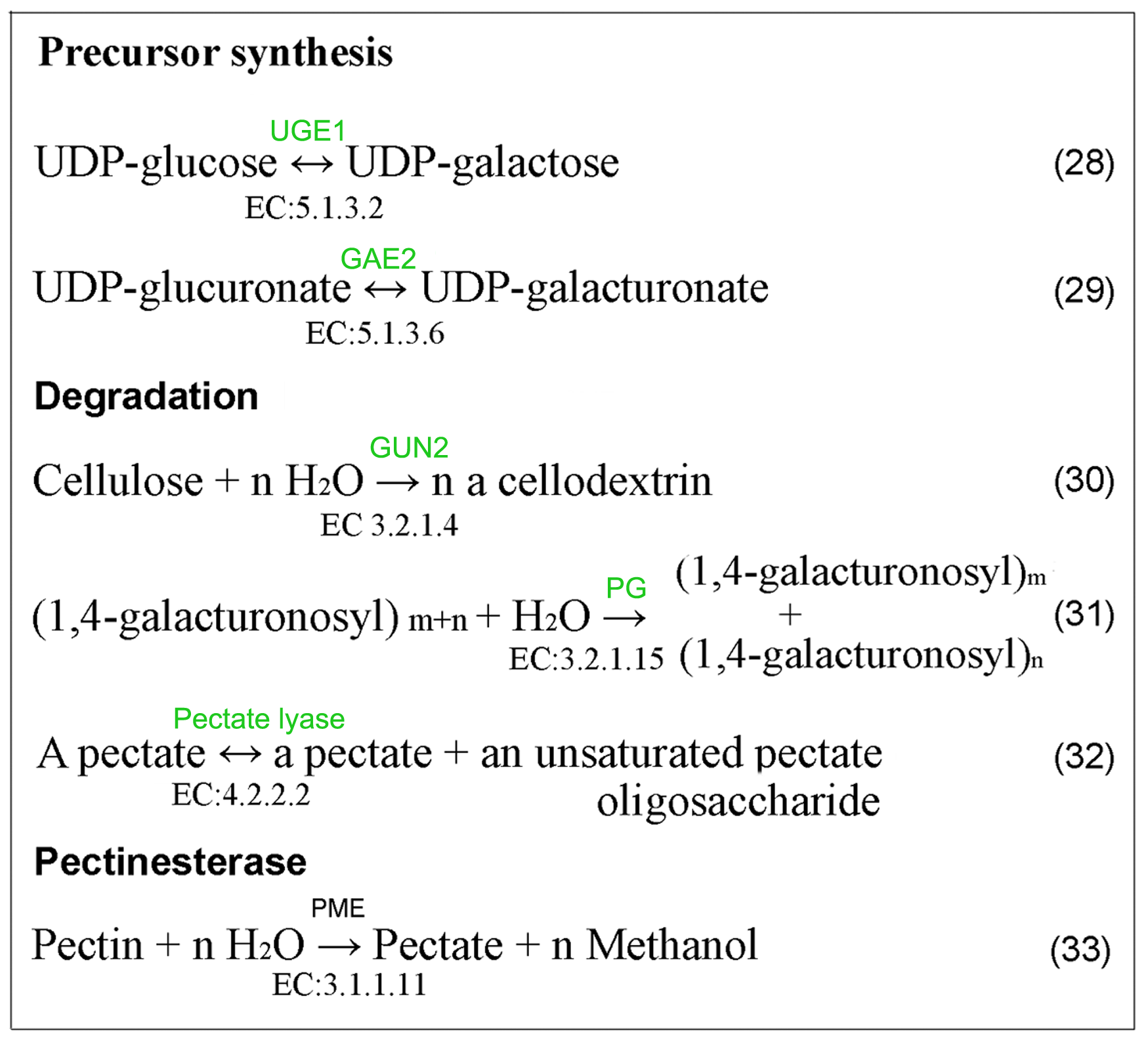

Supplement: S4 Fig — (TIF) [file pone.0182700.s004.tif]

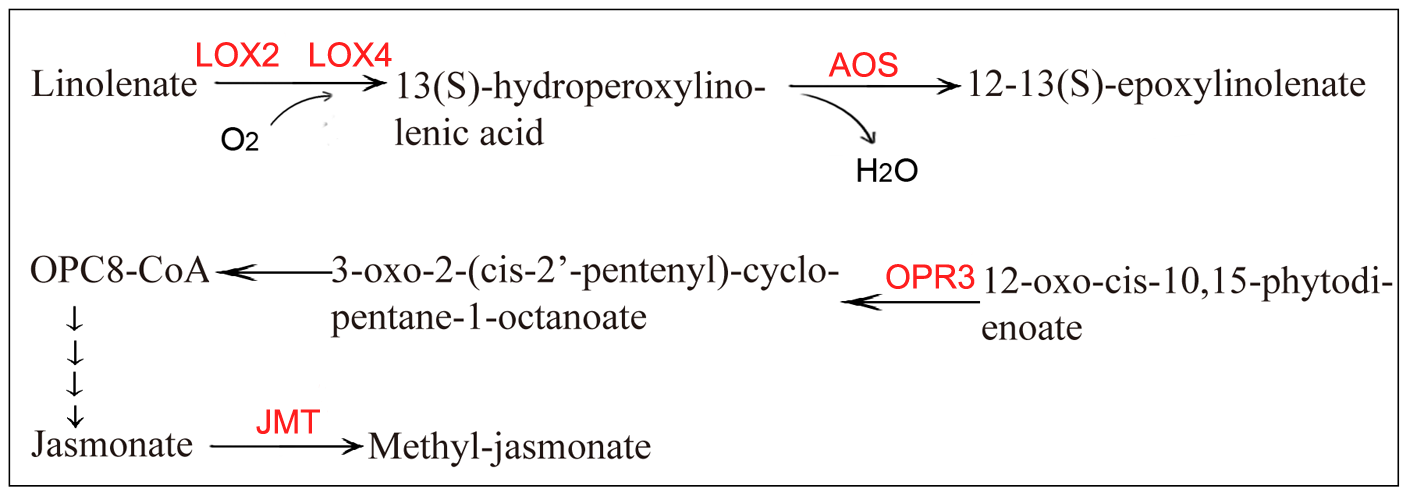

Supplement: S5 Fig — (TIF) [file pone.0182700.s005.tif]

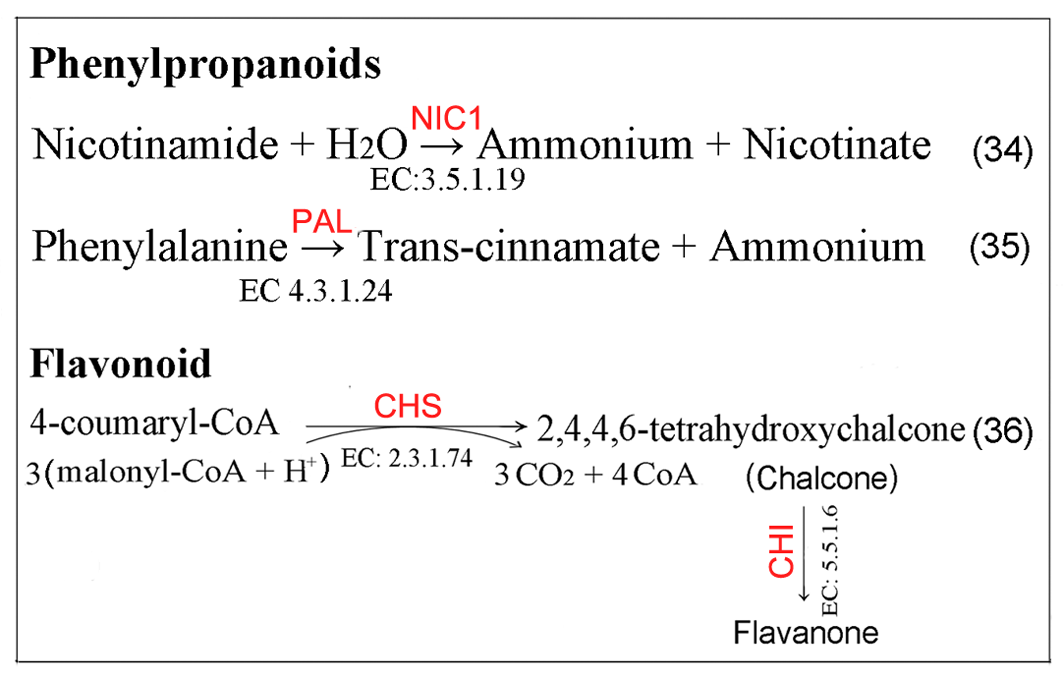

Supplement: S6 Fig — (TIF) [file pone.0182700.s006.tif]
